# Epilepsy insights revealed by intravital functional optical imaging

**DOI:** 10.3389/fneur.2024.1465232

**Published:** 2024-08-29

**Authors:** Matthew A. Stern, Raymond Dingledine, Robert E. Gross, Ken Berglund

**Affiliations:** ^1^Department of Neurosurgery, Emory University School of Medicine, Atlanta, GA, United States; ^2^Department of Pharmacology and Chemical Biology, Emory University School of Medicine, Atlanta, GA, United States; ^3^Department of Neurological Surgery, Rutgers Robert Wood Johnson Medical School, New Brunswick, NJ, United States

**Keywords:** seizure, two-photon, widefield, light sheet, GECI, GEVI, *in vivo* imaging, neurophotonics

## Abstract

Despite an abundance of pharmacologic and surgical epilepsy treatments, there remain millions of patients suffering from poorly controlled seizures. One approach to closing this treatment gap may be found through a deeper mechanistic understanding of the network alterations that underly this aberrant activity. Functional optical imaging in vertebrate models provides powerful advantages to this end, enabling the spatiotemporal acquisition of individual neuron activity patterns across multiple seizures. This coupled with the advent of genetically encoded indicators, be them for specific ions, neurotransmitters or voltage, grants researchers unparalleled access to the intact nervous system. Here, we will review how *in vivo* functional optical imaging in various vertebrate seizure models has advanced our knowledge of seizure dynamics, principally seizure initiation, propagation and termination.

## Introduction

Epilepsy, a serious health condition characterized by recurrent and often disabling seizures (1–3), is the fourth most common neurological disorder (4), with prevalence ranging from 0.5 to 1% around the world (5, 6). Despite over a century of drug development (7), about one third of these cases are medically intractable (8–10). This is perhaps in part due to the approach often taken, agnostic to seizure microcircuitry and network physiology. Surgical interventions such as ablation (11, 12), open resection (13, 14), and electrical stimulation (15, 16) are sometimes options for pharmacoresistant epilepsy. However, none of these procedures render all patients seizure free. Additionally, they can result in off-target effects, and are generally unavailable in developing countries. Thus, a significant need exists for more targeted and effective therapies (17, 18). A deeper understanding of the role of individual neurons, neuronal ensembles, and neural networks in initiating, propagating and terminating epileptic discharges underlying seizures would facilitate such advances.

**Figure 1:**
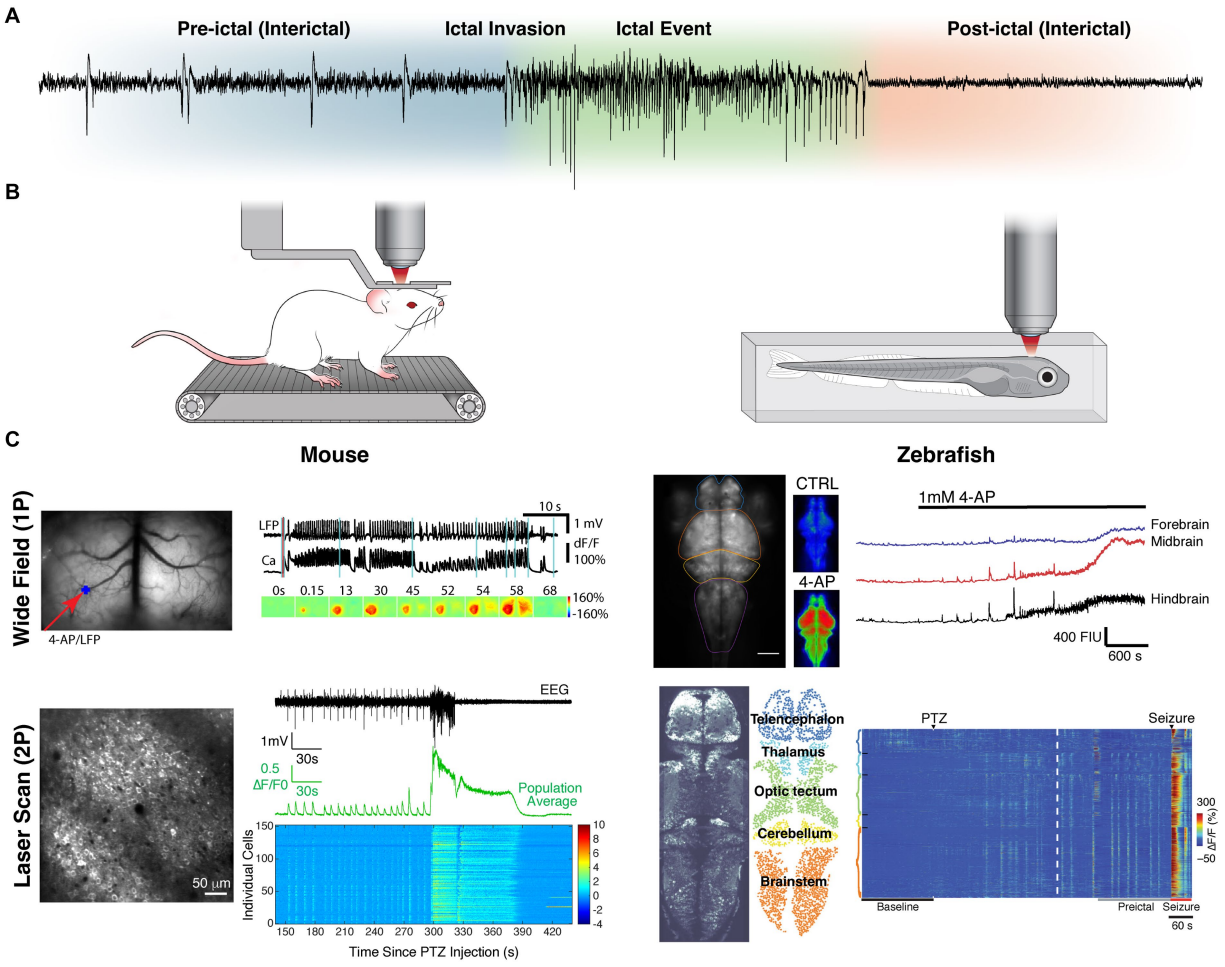
Intravital imaging approaches to studying seizure dynamics in vertebrates. **(A)** Seizure phase partitions with respect to EEG recording during a generalized seizure in a mouse. **(B)** Illustrations of common *in vivo* imaging set-ups (both 1P and 2P). The left depicts a head fixed mouse running on a treadmill and the right depicts a zebrafish embedded in agar. **(C)** Representative fields of view and recordings for 1P (top) and 2P (bottom) imaging in both mouse (left) and zebrafish (right). The mesoscopic spread across brain regions (contiguous ipsilateral and homotopic contralateral in mouse; caudal to rostral in zebrafish) can be appreciated by 1P-widefield imaging. The microscopic individual cell activity (including synchronous pre-ictal firing and local wavefront propagation) can be appreciated with 2P-laser scanning. Data reproduced with modification, under creative commons attribution 4.0 licenses (CC BY) [1P-mouse (116); 2P-mouse (111); 1P-zebrafish (120, 121); 2P-Zebrafish (98)].

Currently, the bulk of our knowledge of seizure dynamics comes from macroelectrode population electrophysiology, with electroencephalography (EEG; Figure 1A) having demonstrated that seizure activity can spread from a focal brain region in a diffuse yet stereotyped network (19). However, this approach fails to capture the complex underlying microcircuit dynamics, many permutations of which can result in the same recorded signal (18). To characterize seizure dissemination between individual neurons, single unit recordings have been performed in humans and animals using microelectrode arrays (MEAs). These recordings have shown that firing and termination of firing of neurons recruited during seizures is highly synchronous and stereotyped (20, 21), where ictal wavefronts propagate with similar directionality across seizures and interictal spikes propagate in antiparallel fashion (22). Additionally, electrophysiology combined with GABAergic pharmacology has been used to find evidence of inhibitory networks restraining seizure activity, including surround and feedforward inhibition (23, 24). However, these recordings have limited recording density, typically at a single cortical depth, due to their planar configuration of sparsely arranged contacts. Furthermore, it is generally impossible to know the subtype of neuron being recorded [for exceptions see (25, 26)]. Moreover, it is challenging to determine if the activity being recorded emanates from the same neuron between separate recording sessions.

Functional optical imaging in animal models (Figure 1B) circumvents many of the limitations of electrophysiology and allows for observation of activity of substantially more neurons. Calcium (27–30) or voltage sensitive dyes (31, 32) were originally used for this purpose, but were rarely cell-type selective, and disadvantaged by significant photobleaching, poor intracellular retention and toxicity (33). The development of genetically encoded indicators, expressed either through viral transduction or through genetic model development, has enabled cell-type specific imaging. These fluorescent proteins undergo a conformational change to excitable states upon the binding of specific ions or small molecules (34, 35). The most commonly used indicators are genetically encoded calcium indicators (GECIs) that fluoresce in the presence of calcium (35–41). They can serve as proxies for neuronal firing, which is marked by an increase of intracellular calcium. In addition to calcium, a growing arsenal of genetically encoded indicators are being developed (34, 42), including for other ions (43–48), small molecules and neurotransmitters (49–58). Furthermore, genetically encoded voltage indicators (GEVIs) have been developed, exploiting voltage-sensitive domains (59–63).

For imaging, these indicators can be excited with either a single photon (1P) in the visible range or two (or more) coincident photons in the infrared range. While 1P excitation can be achieved with many intravital microscope setups, including epifluorescence widefield, laser scanning confocal and light sheet, 2P necessitates the use of a femtosecond mode-locked laser for light delivery (64). Consequently, 2P imaging is limited to laser scanning, although a few light sheet uses exist (65). The temporal and spatial resolution of these methods varies greatly (17, 64, 66–68) and thus the selection of the method should be tailored to the question and indicator. 1P widefield imaging offers the largest fields of view with fastest temporal resolution (Figure 1C). However, for investigations looking at subcellular compartments or multiple cell populations of tens to hundreds of individual neurons, 2P laser scanning is often best. For questions related to brain-wide mesoscale networks, light sheet and 2P laser scanning in transparent zebrafish larva have been the methods most often used. All the above methods require head fixing or immobilizing the subject under an objective. Should the question necessitate a wider range of behavior or longer imaging session, head mounted microscopes [1P (69) and 2P (70, 71)] can be used in freely moving mice. For a more detailed review of epilepsy intravital imaging methodology see (66).

Since the first intravital optical imaging study using a genetically encoded indicator in a vertebrate seizure model nearly a decade ago (72) there have been over 50 additional such studies published. These have sought to examine the contributions of different cell types and neurotransmission to seizure dynamics, the reliability of initiation and propagation patterns, the network changes in synchrony and connectivity that occur at micro and macroscale, and the impact of this aberrant activity on normal function. This review will serve to summarize the major contributions these investigations have provided to our understanding of seizure physiology.

## Overview

Seizures can be partitioned into several distinct phases, namely interictal, pre-ictal, ictal, and post-ictal (Figure 1A). While each phase’s distinct dynamics are of importance, it is the evolution of that activity and the transitions between phases that are often the focus of investigation. Therefore, we have organized our review in a complementary fashion. Table 1 lists the *in vivo* imaging studies included in this review, specifically those that used genetically encoded indicators to investigate epileptiform activity directly. Experiments where imaging was not *in vivo* (e.g., *ex vivo* slice), used dyes, or was only performed during non-epileptiform activity are not discussed.

**Table 1:** Intravital functional optical imaging seizure studies using genetically expressed indicators.

First author	Year	Journal	Method	Species	Model	FOV	Cell type	Indicator type	Seizure phase			
									IIS	Pre-ictal	Ictal	Post-ictal
Deng (54)	2024	Nat Methods	1P-Widefield	Mouse	KA (i.p.)	Ctx	Extracellular	Ca, 5-HT, eCB				•
Jamiolkowski (85)	2024	Nat Med	2P-Laser Scan	Mouse	KA (d. hipp)	FC (Hipp)	FC cells	Ca	•			
Lau (125)	2024	Epilepsia	1P-Miniscope	Mouse	*APP/PS1* Cortical Injury	CA1 (Hipp)	Pan-neuronal	Ca		•	•	
Li (93)	2024	J Cereb Blood Flow Met	1P-Widefield	Mouse	BIC	Ctx	Pyramidal	Ca	•			
Nguyen (127)	2024	Nat Commun	2P-Laser Scan	Mouse	Electrical kindling (hipp), KA (d. hipp)	CA1 (Hipp)	Pan-neuronal, extracellular	Ca, ACh			•	
Shah (114)	2024	Cell Rep	1P-Widefield, 2P-Laser Scan	Mouse	4-AP	Ctx (II/III)	Pan-neuronal + Nkx2.1 (PV, SST) reporter	Ca	•		•	
Stern (111)	2024	Neurophotonics	2P-Laser Scan	Mouse	PTZ	Ctx (II/III)	VGAT + non-VGAT	Ca		•	•	•
Burrows (105)	2023	J Neurosci	2P-Laser Scan	Zebrafish	PTZ	WB	Pan-neuronal	Ca		•	•	
Li (88)	2023	iScience	2P-Laser Scan	Mouse	*scn2a* + PTZ	Ctx (V)	Pyramidal	Ca	•			
Luo (77)	2023	Epilepsia	1P-Widefield	Mouse	BIC	Ctx	Pyramidal, PV	Ca	•			
Masala (82)	2023	Brain	2P-Laser Scan	Mouse	KA (d. hipp)	CA1 (Hipp)	Pyramidal	Ca	•			
Shimoda (91)	2023	Brain	2p-Spiral Linescan	Mouse	4-AP, Ptx	Ctx (I)	Extracellular	GABA, Glu	•	•		
de Vito (118)	2022	Biomed Opt Express	2P-Light Sheet, 1P-Widefield	Zebrafish	PTZ	WB	Pan-neuronal	Ca			•	•
Dong (56)	2022	Nat Biotechnol	2P-Laser Scan	Mouse	Electrical kindling (v. hipp)	CA1 (Hipp)	Pan-neuronal, extracellular	Ca, eCB			•	•
Hotz (100)	2022	Glia	1P-Widefield, 2P-Laser Scan	Zebrafish	PTZ, *eaat2a* + photostim	WB	Pan-neuronal, astroglial	Ca, Glu		•	•	
Mulcahey (108)	2022	eNeuro	2P-Laser Scan + Transparent MEA	Mouse	4-AP	CA1 (Hipp)	Pyramidal	Ca			•	
Myren-Svelstad (101)	2022	Epilepsia	2P-Laser Scan	Zebrafish	PTZ, *eaat2a*, *gabra1* + photostim	WB	Pan-neuronal, astroglial	Ca		•	•	•
Niemeyer (80)	2022	Brain	2P-Laser Scan	Zebrafish	PTZ	WB	Pan-neuronal + VGLUT2 reporter	Ca	•	•	•	
Özsoy (119)	2022	Front Mol Neurosci	1P-Widefield, Photoacoustic	Zebrafish	*eaat2a* + photostim	WB	Pan-neuronal	Ca			•	
Turrini (99)	2022	Biomedicines	2P-Light Sheet	Zebrafish	PTZ	WB	Pan-neuronal	Ca		•	•	•
Zhang (109)	2022	Neurosci Bull	2P-Miniscope	Mouse	KA (i.p.)	Ctx*	Pan-neuronal	Ca		•	•	•
Bando (90)	2021	Nat Commun	2P-Laser Scan	Mouse	4-AP	Ctx (I-VI)	Pyramidal	Ca, Voltage	•	•	•	
Driscoll (107)	2021	Commun Biol	1P-Widefield + Transparent MEA	Mouse	4-AP	Ctx	Pyramidal	Ca			•	
Farrell (126)	2021	Neuron	2P-Laser Scan	Mouse	Electrical kindling (v. hipp)	CA1 (Hipp)	Pan-neuronal, extracellular	Ca, eCB			•	
Hadjiabadi (84)	2021	Neuron	2P-Laser Scan	Zebrafish; Mouse	PTZ; KA (v. hipp)	WB; DG (Hipp)	Pan-neuronal; GCs + abGC reporter	Ca	•	•	•	
Lim (103)	2021	J Cereb Blood Flow Met	2P-Laser Scan	Mouse	4-AP	Ctx (II/III)	Pyramidal, GABAergic	Ca		•	•	
Liu (79)	2021	iScience	1P-Spinning disc	Zebrafish	*stxbp1p*	WB	Pan-neuronal	Ca	•			
Somarowthu (115)	2021	Cell Calcium	2P-Laser Scan	Mouse	*scn1a* + Heat	Ctx*	Pan-neuronal + PV, SST, VIP reporters	Ca		•	•	
Wong (110)	2021	Neuropsychopharmacology	1P-Miniscope	Mouse	*scn8a* + PTZ	Ctx	Pyramidal	Ca			•	
Yang (116)	2021	Front Neurosci	1P-Widefield	Mouse	4-AP	Ctx	Pyramidal	Ca			•	•
Aeed (89)	2020	Ann Neurol	2P-Laser Scan	Mouse	4-AP	Ctx (II/III, V)	Pyramidal; PV; SST (separate)	Ca	•	•	•	
Cozzolino (78)	2020	Cells	2P-Laser Scan	Zebrafish	PTZ; *kcnj10a*	WB	Pan-neuronal	Ca	•	•		
Farrell (124)	2020	Sci Rep	2P-Laser Scan	Mouse	Electrical kindling (v. hipp)	CA1 (Hipp)	Pyramidal	Ca			•	•
Hatcher (117)	2020	J Clin Invest	1P-Widefield	Mouse	Glioma	Ctx	Pan-neuronal	Ca			•	•
Montgomery (76)	2020	Cell Rep	1P-Widefield, 2P-Laser Scan	Mouse	Glioma	Ctx	Pyramidal	Ca	•		•	
Shuman (94)	2020	Nat Neurosci	1P-Miniscope	Mouse	Pilo (I.P.)	CA1 (Hipp)	Pan-neuronal	Ca	•			
Sparks (83)	2020	Nat Commun	2P-Laser Scan	Mouse	KA (v. hipp)	DG (Hipp)	Pan-neuronal + abGC reporter	Ca	•			
Tran (131)	2020	JCI Insight	2P-Laser Scan	Mouse	Max electroshock	Ctx*	Astroglial, mural	Ca				•
Tran (102)	2020	J Neurosci	2P-Laser Scan	Mouse	*scn1a* + Heat	Ctx (II/III)	Pan-neuronal + PV reporter	Ca		•	•	•
Brenet (96)	2019	Cells	1P-Widefield	Zebrafish	*scn1a*	WB	Pan-neuronal	Ca			•	
Jayant (112)	2019	Cell Rep	2P-Laser Scan + Nanopipette	Mouse	4-AP	Ctx (II/III)	Pyramidal	Ca		•	•	
Liao (97)	2019	Dis Model Mech	1P-Spinning Disc; 1P-Light Sheet	Zebrafish	*gabrg2* + photostim	WB	Pan-neuronal	Ca			•	
Liu (81)	2019	eNeuro	2P-Laser Scan	Zebrafish	PTZ, 4-AP	WB	Pan-neuronal	Ca	•	•	•	
Marvin (51)	2019	Nat Methods	2P-Laser Scan	Mouse	Pilo (ctx)	Ctx (II/III)	Extracellular	GABA	•			
Verdugo (98)	2019	Nat Commun	2P-Laser Scan	Zebrafish	PTZ	WB	Pan-neuronal, astroglial, extracellular	Ca, Glu		•	•	
Wenzel (95)	2019	J Neurosci	2P-Laser Scan	Mouse	4-AP	Ctx (II/III)	Pan-neuronal + PV reporter	Ca		•	•	
Heuser (86)	2018	Cereb Cortex	2P-Laser Scan	Mouse	KA (i.p.)	CA1 (Hipp)	Pan-neuronal, astroglial	Ca	•		•	•
Liou (23)	2018	Brain	2P-Laser Scan	Mouse	4-AP	Ctx (II/III)	PV	Ca		•		
Meyer (122)	2018	Nat Commun	2P-Laser Scan	Mouse	*stargazer*	Ctx (II/III-VI)	Pan-neuronal + IHC reporters	Ca		•	•	
Rosch (104)	2018	PLoS Comput Biol	1P-Light Sheet	Zebrafish	PTZ	WB	Pan-neuronal	Ca		•		
Zhang (106)	2018	Nano Lett	2P-Laser Scan + transparent MEA	Mouse	4-AP	Ctx*	Pyramidal	Ca			•	
Petrucco (87)	2017	Sci Rep	2P-Laser Scan	Mouse	BIC	Ctx (II/III)	Pyramidal	Ca	•			
Rossi (74)	2017	Nat Commun	1P-Widefield	Mouse	Pilo, Ptx	Ctx	Pan-neuronal, pyramidal	Ca	•		•	
Sato (44)	2017	Proc Natl Acad Sci	2P-Laser Scan	Mouse	4-AP	Ctx (II/III)	CAG-promoter	Cl, pH			•	•
Steinmetz (75)	2017	eNeuro	1P-Widefield	Mouse	various transgenics	Ctx	various	Ca	•			
Turrini (120)	2017	Sci Rep	1P-Widefield	Zebrafish	PTZ	WB	Pan-neuronal	Ca		•	•	
Wenzel (113)	2017	Cell Rep	2P-Laser Scan	Mouse	4-AP, Pilo	Ctx (II/III, V)	Pan-neuronal	Ca			•	
Winter (121)	2017	Sci Rep	1P-Light Sheet, 1P-Widefield	Zebrafish	PTZ, 4-AP, Pilo, Strychnine	WB	Pan-neuronal	Ca			•	
Berdyyeva (123)	2016	Front Neurosci	1P-Miniscope	Mouse	KA (i.p.); NMDA; PTZ	CA1 (Hipp)	Pyramidal	Ca			•	•
Muldoon (72)	2015	Brain	2P-Laser Scan	Mouse	Pilo	CA1 (Hipp)	Pan-neuronal, GABAergic	Ca	•			

1P, one-photon; 2P, two-photon; 4-AP, 4-aminopyridine; 5-HT, 5-hydroxytryptamine (serotonin); BIC, bicuculline; Ca, calcium; Ctx, cortex; DG, dentate gyrus; eCB, endocannabinoid; FC, fasciola cinereum; GABA, gamma-aminobutyric acid; GC, granule cell (ab, adult born; m, mature); Glu, glutamate; Hipp, hippocampus (d, dorsal; v, ventral); IHC, immunohistochemistry; i.p., intraperitoneal; KA, kainic acid; NMDA, N-methyl-D-aspartate; Pilo, pilocarpine; Ptx, picrotoxin, PTZ, pentylenetetrazol; PV, parvalbumin; SST, somatostatin; VGAT, vesicular GABA transporter; VGLUT, vesicular glutamate transporter; VIP, vasoactive intestinal peptide; WB, whole brain; *Imaging depth/layer not reported; Italics indicate transgenic line.

## Interictal activity

Interictal spikes (IIS) are episodes of transient synchronous paroxysmal depolarization across ensembles of hyperexcitable neurons, classically observed as a spike wave discharge on EEG (73). Widefield imaging of the cortex has demonstrated that IIS begin as standing waves in local regions with limited contiguous spread (74), although some cortex-wide propagation and delayed recruitment of distal non-contiguous foci has been observed (75, 76). While IIS are often limited to or emanating from the purported epileptic focus, in instances of extrafocal origin, or non-contiguous spread, these loci often share homotopic connection with the ictal focus (74, 77). These patterns have been observed in both excitatory and inhibitory populations (77). The local and limited nature of interictal activity is corroborated by whole brain imaging in zebrafish (78–80).

To parse the individual cell activity patterns underlying IIS, laser scanning microscopy was employed. Imaging the zebrafish optic tectum revealed hypersynchronous recruitment of microensembles underlying the spatially confined interictal activity observed by previous studies with widefield microscopy (79, 81). In the pilocarpine chronic seizure mouse model, it was shown that inhibitory neurons are disproportionally active relative to pyramidal cells during IIS in CA1, consistent with a perisomatic inhibitory restraint occurring. Additionally, while it seems there are subpopulations of neurons consistently active together, their recruitment is varied across spikes (72). However, in a chronic intrahippocampal kainic acid (KA) mouse model, synchronous bursts of pyramidal cells have been observed, which could speak to model differences in ictogenic mechanisms (82). When imaging the dentate gyrus (DG) also following intrahippocampal KA, distinct microensembles of excitatory adult born granule cells (abGCs) were determined to overly drive IIS, albeit firing in a desynchronized manner. The specific ensembles recruited across IIS were varied. These are distinct from the microensembles that participate in sharp wave ripples, which were shown to be driven by both mature and abGCs, firing with greater synchrony. This suggests that decoupling of abGCs from mature GCs and subsequent reorganization into these desynchronized pathologic ensembles may contribute to an impairment in dentate gating, enabling ictogenesis (83). When training computational models on calcium data from this model, abGCs were most often identified as superhub neurons with high feedforward conductance, enhancing downstream excitation in the resulting epileptic networks (84). Neurons outside the hippocampus, but still part of this network have also been found to be involved in seizure regulation, such as the fasciola cinereum, a collateral intermediary nucleus connecting the entorhinal cortex to the DG (85). Gap junctions may be in part mediating the spread of IIS activity, particularly in the astrocyte syncytium, as blocking gap junctions significantly decreased the occurrence, duration and spread of IIS (79). However, while astrocytes in CA1 exhibit transient increases in calcium spontaneously during the interictal period, these have been observed asynchronous with IIS (86).

For those spikes occurring in the cortex there is a notable recruitment of pyramidal cells (87, 88), limited to layer II/III, in addition to inhibitory cells (89). While both parvalbumin (PV) and somatostatin (SST) cells demonstrate activity during IIS, PV cells were predominantly recruited with a higher degree of inter- (with pyramidal cells) and intrapopulation synchrony, while SST cells demonstrated asynchronous and delayed recruitment (89). Combined GEVI and GECI imaging in pyramidal cells revealed that there is little supra- or subthreshold activity propagating out of the focus during IIS (90). Studies utilizing neurotransmitter indicators were concordant with these IIS dynamics, revealing an increase in glutamate observed at the focus, which expanded centrifugally, and an increase in gamma-aminobutyric acid (GABA) observed extrafocally, which displayed slower and more persistent centripetal propagation, consistent with intact feedforward inhibitory surround limiting the IIS spread (51, 91). Indeed, the IIS may reflect this restraining mechanism (92). Importantly, the majority of these studies were performed in focal neocortical models, where a chemoconvulsant was intracortically injected to elicit epileptiform activity. Corroborating studies across other models could strengthen the generalizability of these findings.

Combined widefield GECI imaging with optical imaging of intrinsic hemodynamic signal to examine neurovascular coupling showed that during IIS there is an initial ‘epileptic’ dip in hemoglobin oxygenation, likely the result of vasodilation, followed by a period of hyperoxygenation, due to increased levels of total hemoglobin delivery, all of which is tightly spatially correlated with excitatory cell activation (76, 93).

In terms of functional impact, hippocampal place cell encoding is impaired in two chronic seizure models, with aberrant dendritic hyperexcitability (82) and aberrant firing and desynchronization (94) hypothesized as contributing mechanisms.

Taken together, intravital imaging at microcircuit resolution has revealed distinct cell type activity patterns specific to different anatomical regions during IIS, and speaks also to intra- vs. extrafocal differences (92). There is considerable variability in the specific neurons recruited, where different ensembles can be recruited across sequential IISs.

## Pre-ictal to ictal transition

Interictal periods, by definition, occur between seizures and thus they have both pre-ictal and post-ictal phases, the exact boundaries of which are poorly defined. Thus, to examine the pre-ictal phase, we look at the interictal period from the perspective of the progression of dynamic changes as the brain state transitions to seizure.

An advantage of imaging is the ability to delineate activity as intra- or extrafocal. When recording from the focus in a mouse 4-aminopyridine (4-AP) model, recruitment of both excitatory and inhibitory neurons in local microensembles, akin to microseizures, was observed prior to ictal onset. As the brain progressed to seizure, ensemble activity increased across populations, with synchronization amongst excitatory cells (95). When examining dynamics at mesoscales in zebrafish whole brain, the mesencephalon/optic tectum, and occasionally the thalamus, emerged as a conserved region of hyperactivity (96–98) and seizure focus (78, 80, 81, 99–101). It was found that foci tended to have a higher proportion of excitatory cells than in the penumbra, perhaps contributing to their hyperexcitability (80).

When exploring cortical regions outside of the seizure onset zone prior to seizure invasion, feedforward inhibitory activity was observed, be it increased firing activity of inhibitory neurons (23, 95, 102, 103), or elevated GABA release during pre-ictal spikes relative to within the focus (91). Excitatory recruitment was still discernable, although possibly with some degree of suppression (95). As the tissue transitioned to a seizure state, there was a progressive increase in synchronization amongst pyramidal cells (89, 102), with a gradual breakdown in the inhibitory surround, witnessed as desynchronization in the PV cells activity (89, 102) and a decrease in released GABA during spiking (91). Inhibitory restraint weakening is also detected as progressively increasing bursts of subthreshold activity in pyramidal cells by GEVI imaging in the penumbra (90). As compared with neurons, astroglial networks displayed a more widespread elevation in calcium activity and synchrony pre-ictally, although their bursts of activity seemed to follow immediately after neuronal bursts during pre-ictal spikes (98).

Modeling based upon whole brain zebrafish mesoscale 1P light sheet imaging during PTZ induced seizures found that the tectum served as a networkwide hub. As the brain transitioned to seizure, there was a decrease in input to the tectum, enabling downstream network synchronization. The brain-wide recruitment was facilitated by increasing faster excitatory transmission and decreasing slower inhibitory transmission (104). Similar findings emerged from a model trained on 2P data with single-cell resolution collected from the same model. Specifically, they found that pre-ictal networks had enriched feedforward motif conductance, especially amongst “superhub” neurons, which promoted the pro-seizure tendency of the network (84). Another computational study using 2P data in the same model estimated microscale avalanche dynamics and showed that there was an increase in network connectivity at single-cell resolution, which drove the brain away from criticality, a point of maximal flexibility in brain state, thus limiting phase transition possibilities until the system converged on an inflexible ictal state (105).

## Seizure propagation and ictal dynamics

Upon seizure invasion, suprathreshold activity is observed, first as a fast voltage wave and then a slower calcium wave (0.5–1 s delay) (90). Simultaneous calcium imaging through transparent microelectrode and electrocorticography arrays demonstrate spatial concordance between the modalities (106), where progression of ictal electrophysiology is tied with expansion of ictal core (107, 108).

Highly elevated and sustained calcium can be detected (109, 110) in both excitatory cells and inhibitory neurons (95, 102, 111), with pyramidal cells displaying the greatest recruitment and hypersynchrony (80, 89, 103). A concordant expansion of glutamate release into the field is also observed (91, 100), hypothesized to be in part released by glia (98). However, while calcium amplitude is classically thought to be directly correlated with activity, a large increase could also indicate intracellular calcium homeostasis breakdown, and thus an absence of firing. A simultaneous *in vivo* imaging and patch clamp study revealed that the PV cells with an ictal calcium increase, actually enter a state of depolarization block upon seizure invasion (112), consistent with inhibitory restraint collapse.

Propagation can be witnessed as a calcium wavefront (102, 111, 113), yet the recruitment of interneurons within this seems variable. In the 4-AP model the PV neuron recruitment appears spatially heterogenous (95) at invasion, with a delayed recruitment in SST cells (89). However, in a study imaging Nkx2.1 cells (PV and SST), these were found to be recruited in a spatiotemporally concordant manner to the other cells in the field (114). In a Dravet mouse model with thermally induced seizures, PV and vasoactive intestinal peptide-expressing (VIP) cells appear recruited along with the population, while SST cells demonstrate a spatially independent early recruitment (115). With respect to cortical layers, layer II/III pyramidal cells tend to be the first to propagate during the seizure, with a lagging recruitment of layer V (89, 113). The propagation has also been found to have reliable recruitment across sequential ictal events following intracortical injection of a chemoconvulsant (95, 113). The speed of propagation varied across events, although when the cortex had been first disinhibited by picrotoxin, the speed increased and variability was dampened, consistent with the inhibitory restraint hypothesis (113). Interestingly, in a study where optogenetic photostimulation during calcium imaging was used to determine excitability in individual cells, decreased excitability proximal to the invading seizure wavefront was observed, in contrast to hyperexcitability observed interictally and baseline level excitability observed distal to invasion, suggesting that inhibitory neurons recruited to the seizure generate a front of local inhibition (114).

Propagation through brain regions appears to follow both proximal contiguous and distal homotopic spread with respect to the onset zone (74), including contralateral projections (76, 116, 117). In zebrafish, whole brain propagation was found to typically occur caudal to rostral (78, 100, 118, 119), although the opposite direction was occasionally observed (81), perhaps related to the different developmental stage and chemoconvulsant dosage used (118). While activity in rostral regions (telencephalon and habenula) is not initially correlated with the caudal regions (optic tectum, cerebellum and medulla), entrainment occurs along the rostrocaudal axis upon progression to seizure (98, 99, 120). Indeed, eventually the brain-wide synchrony can be observed (81, 84, 97, 104, 118), although there is some model dependency on the exact extent of recruitment (101, 121). The astroglial syncytium’s calcium activity also displays brain-wide hyperactivity (100) and synchrony within itself and with neurons during seizures (98). There is, though, a short delay in the astroglial ictal recruitment (101), corresponding to a further increase in neural activity, consistent with the hypothesis that neural activity is exacerbated by glial glutamate release (98). Impaired glutamate reuptake by astrocytes also led to hyperexcitability with spontaneous seizures and concurrent excessive glutamate signal (100). At seizure invasion, there is a large increase in intracellular chloride that slowly builds throughout the seizure, while pH slightly decreases (44).

Interestingly, imaging in the visual cortex of a mouse absence model revealed an opposite finding to the other seizure models, a decrease in neuronal activity and synchronization across cortical layers and neuron subtypes during ictal episodes. This asynchronous suppression could be related to the impaired visual awareness classically associated with this seizure type (122).

In the hippocampus, ictal activity shows recruitment in both CA1 pyramidal cells (123, 124), as well as the fasciola cinereum (85). While recruitment is highly synchronous for many neurons at invasion, new neurons are continuously recruited throughout the seizure (125). Spatial propagation dynamics recapitulated those of the cortex, demonstrating within-subject reliability for sequential events and a much faster expansion of electrophysiologic signatures ahead of the neuronal recruitment to the propagating calcium wave (108). Additionally, spatiotemporally concordant release of endocannabinoids occurs, which may play a feedback role in restricting seizure activity (56, 126). Acetylcholine levels have also been shown to increase during seizures, strongly correlating with intracellular calcium (127). Astrocytic calcium increased during seizure invasion as well, sometimes preceding the event, which may be in part mediated by internal store release (86).

With respect to neurovascular coupling, while the ictal focus is typically well supplied and only occasionally hypoxic, the penumbra is hyperoxygenated during seizure initiation and propagation, extending beyond the recruited tissue. At initiation, the blood supply to the focus increases, and this expansion appears as a wave ahead of the calcium wave in neurons into the penumbra and persists into the post-ictal phase (76, 116).

## Seizure termination and post-ictal activity

Upon seizure termination or shortly into the post-ictal period, slow propagating waves of calcium have been recorded in the cortex (54, 102, 109, 111, 117) and hippocampus (56, 86, 123, 124). These waves have been hypothesized to be spreading depolarizations as they share similar spatiotemporal propagation properties (128), and spreading depolarizations can be temporally associated with seizures (129, 130). Spatiotemporally concordant serotonin and endocannabinoids waves have also been detected during these calcium waves (54, 56). These calcium waves are then followed by periods of post-ictal suppression of activity and synchrony (118, 124), with one zebrafish study showing functional connectivity to bifurcate into rostral and caudal groups (99). Intracellular chloride returns to baseline levels upon seizure termination and a gradual intracellular acidification occurs post-ictally (44).

A transient increase in astrocytic calcium was also observed post-ictally (86), corresponding to post-ictal vasoconstriction (131), as well as post-ictal hypoactivity (101). While this glial calcium level was sustained for at most a few minutes, vasoconstriction was observed for over an hour. Vasculature smooth muscle cells also showed elevated calcium for the duration of the vasoconstriction (131). When post-ictal vasoconstriction was depressed by a COX-2 inhibitor, the duration of astrocytic calcium elevation was significantly diminished (131), while post-ictal suppression or recovery of neural activity was unchanged (124). On the other hand, when glia glutamate reuptake was impaired, post-ictal hypoactivity was diminished (101). Taken together, a vascular coupling to neuronal and glial activity is present post-ictally, although there may be a dissociation from their post-ictal suppressive mechanisms.

## Looking forward

Intravital microscopy coupled with genetically targeted indicators has allowed unprecedented access to the intact nervous system. Leveraging these powerful tools across a variety of vertebrate seizure models has provided deep insight into mechanisms of epilepsy. Imaging has confirmed previous hypotheses derived from electrophysiology, such as the existence of microseizures inside an epileptic focus (132, 133) and the role of inhibition in restraining seizure activity (19, 24, 134). Imaging has also enabled new discoveries which have opened possibilities for novel treatment targets and approaches to epilepsy (17, 135), such as the neuromodulation of adult-born dentate granule cell superhubs (84), or leveraging seizure specific neurochemical changes for drug design (127), which could be extended as an autoregulatory gene therapy (136). Optical and genetic technologies are advancing quickly, opening even more possibilities. Already there are methods that could allow imaging of the whole cortex at a single cell resolution (137, 138), that enable imaging deeper in the brain without the need to aspirate the cortex (139), that capture activity in freely moving subjects to better tie behavior to ensemble activity (119, 140), and to decode electrophysiologic population dynamics from microensemble activity (106, 107).

Collectively, functional optical imaging modalities have immense scalability, to image at the micro-, meso- and macro-circuit level, allowing inference to be drawn about the interplay between the cellular and network evolution of seizures (17, 18, 66). While we think about a classic seizure as evolving, massive hypersynchronous activity, largely due to the use of EEG to identify and study these dynamics, functional optical imaging has made it abundantly clear that while that certainly is a defining feature, there are intricate activity patterns across cell types precipitating and underlying these events.

## References

[1] Chadwick D Taylor J Johnson T. Outcomes after seizure recurrence in people with well-controlled epilepsy and the factors that influence it. Epilepsia. (1996) 37:1043–50. doi: 10.1111/j.1528-1157.1996.tb01023.x 8917053

[2] Hauser WA Rich SS Lee JRJ Annegers JF Anderson VE. Risk of recurrent seizures after two unprovoked seizures. N Engl J Med. (1998) 338:429–34. doi: 10.1056/NEJM199802123380704 9459646

[3] Fisher RS Acevedo C Arzimanoglou A Bogacz A Cross JH Elger CE. ILAE official report: a practical clinical definition of epilepsy. Epilepsia. (2014) 55:475–82. doi: 10.1111/epi.12550, PMID: 24730690

[4] Hirtz D Thurman DJ Gwinn-Hardy K Mohamed M Chaudhuri AR Zalutsky R. How common are the "common" neurologic disorders? Neurology. (2007) 68:326–37. doi: 10.1212/01.wnl.0000252807.38124.a3 17261678

[5] Zack MM Kobau R. National and state estimates of the numbers of adults and children with active epilepsy - United States, 2015. Morbidity Mortality Weekly Rep. (2017) 66:821–5. doi: 10.15585/mmwr.mm6631a1, PMID: 28796763 PMC5687788

[6] Beghi E Giussani G Abd-Allah F Abdela J Abdelalim A Abraha HN. Global, regional, and national burden of epilepsy, 1990-2016: a systematic analysis for the global burden of disease study 2016. Lancet Neurol. (2019) 18:357–75. doi: 10.1016/S1474-4422(18)30454-X, PMID: 30773428 PMC6416168

[7] Rho JM White HS. Brief history of anti-seizure drug development. Epilepsia Open. (2018) 3:114–9. doi: 10.1002/epi4.12268 30564769 PMC6293064

[8] Hauser WA Kurland LT. Epidemiology of epilepsy in Rochester, Minnesota, 1935 through 1967. Epilepsia. (1975) 16:1–66. doi: 10.1111/j.1528-1157.1975.tb04721.x, PMID: 804401

[9] Kwan P Brodie MJ. Early identification of refractory epilepsy. N Engl J Med. (2000) 342:314–9. doi: 10.1056/NEJM200002033420503 10660394

[10] Kwan P Sperling MR. Refractory seizures: try additional antiepileptic drugs (after two have failed) or go directly to early surgery evaluation? Epilepsia. (2009) 50:57–62. doi: 10.1111/j.1528-1167.2009.02237.x, PMID: 19702735

[11] Gross RE Stern MA Willie JT Fasano RE Saindane AM Soares BP. Stereotactic laser amygdalohippocampotomy for mesial temporal lobe epilepsy. Ann Neurol. (2018) 83:575–87. doi: 10.1002/ana.25180, PMID: 29420840 PMC5877322

[12] Wu CY Jermakowicz WJ Chakravorti S Cajigas I Sharan AD Jagid JR. Effects of surgical targeting in laser interstitial thermal therapy for mesial temporal lobe epilepsy: a multicenter study of 234 patients. Epilepsia. (2019) 60:1171–83. doi: 10.1111/epi.15565, PMID: 31112302 PMC6551254

[13] Wiebe S Blume WT Girvin JP Eliasziw M Effectiveness and Efficiency of Surgery for Temporal Lobe Epilepsy Study Group. A randomized, controlled trial of surgery for temporal-lobe epilepsy. N Engl J Med. (2001) 345:311–8. doi: 10.1056/NEJM200108023450501 11484687

[14] Josephson CB Dykeman J Fiest KM Liu X Sadler RM Jette N. Systematic review and meta-analysis of standard vs selective temporal lobe epilepsy surgery. Neurology. (2013) 80:1669–76. doi: 10.1212/WNL.0b013e3182904f82, PMID: 23553475

[15] Salanova V Witt T Worth R Henry TR Gross RE Nazzaro JM. Long-term efficacy and safety of thalamic stimulation for drug-resistant partial epilepsy. Neurology. (2015) 84:1017–25. doi: 10.1212/WNL.0000000000001334, PMID: 25663221 PMC4352097

[16] Heck CN King-Stephens D Massey AD Nair DR Jobst BC Barkley GL. Two-year seizure reduction in adults with medically intractable partial onset epilepsy treated with responsive neurostimulation: final results of the RNS system pivotal trial. Epilepsia. (2014) 55:432–41. doi: 10.1111/epi.12534, PMID: 24621228 PMC4233950

[17] Rossi LF Kullmann DM Wykes RC. The enlightened brain: novel imaging methods focus on epileptic networks at multiple scales. Front Cell Neurosci. (2018) 12:1–8. doi: 10.3389/fncel.2018.00082 29632475 PMC5879108

[18] Farrell JS Nguyen QA Soltesz I. Resolving the Micro-macro disconnect to address Core features of seizure networks. Neuron. (2019) 101:1016–28. doi: 10.1016/j.neuron.2019.01.043, PMID: 30897354 PMC6430140

[19] Schevon CA Weiss SA McKhann G Goodman RR Yuste R Emerson RG. Evidence of an inhibitory restraint of seizure activity in humans. Nat Commun. (2012) 3:1060. doi: 10.1038/ncomms2056, PMID: 22968706 PMC3658011

[20] Truccolo W Donoghue JA Hochberg LR Eskandar EN Madsen JR Anderson WS. Single-neuron dynamics in human focal epilepsy. Nat Neurosci. (2011) 14:635–41. doi: 10.1038/nn.2782, PMID: 21441925 PMC3134302

[21] Truccolo W Ahmed OJ Harrison MT Eskandar EN Cosgrove GR Madsen JR. Neuronal ensemble synchrony during human focal seizures. J Neurosci. (2014) 34:9927–44. doi: 10.1523/JNEUROSCI.4567-13.2014 25057195 PMC4107409

[22] Smith EH Liou YY Merricks EM Davis T Thomson K Greger B. Human interictal epileptiform discharges are bidirectional traveling waves echoing ictal discharges. Elife. (2022) 11:20. doi: 10.7554/eLife.73541 PMC8813051 35050851

[23] Liou JY Ma HT Wenzel M Zhao MR Baird-Daniel E Smith EH. Role of inhibitory control in modulating focal seizure spread. Brain. (2018) 141:2083–97. doi: 10.1093/brain/awy116, PMID: 29757347 PMC6022627

[24] Trevelyan AJ Schevon CA. How inhibition influences seizure propagation. Neuropharmacology. (2013) 69:45–54. doi: 10.1016/j.neuropharm.2012.06.015 22722026

[25] Neumann AR Raedt R Steenland HW Sprengers M Bzymek K Navratilova Z. Involvement of fast-spiking cells in ictal sequences during spontaneous seizures in rats with chronic temporal lobe epilepsy. Brain. (2017) 140:2355–69. doi: 10.1093/brain/awx179, PMID: 29050390 PMC6248724

[26] Miri ML Vinck M Pant R Cardin JA. Altered hippocampal interneuron activity precedes ictal onset. eLife. (2018) 7:20. doi: 10.7554/eLife.40750 PMC6245730 30387711

[27] Trevelyan AJ Sussillo D Watson BO Yuste R. Modular propagation of epileptiform activity: evidence for an inhibitory veto in neocortex. J Neurosci. (2006) 26:12447–55. doi: 10.1523/JNEUROSCI.2787-06.2006, PMID: 17135406 PMC6674895

[28] Trevelyan AJ Sussillo D Yuste R. Feedforward inhibition contributes to the control of epileptiform propagation speed. J Neurosci. (2007) 27:3383–7. doi: 10.1523/JNEUROSCI.0145-07.2007, PMID: 17392454 PMC6672122

[29] Baird-Daniel E Daniel AGS Wenzel M Li D Liou JY Laffont P. Glial calcium waves are triggered by seizure activity and not essential for initiating ictal onset or neurovascular coupling. Cereb Cortex. (2017) 27:3318–30. doi: 10.1093/cercor/bhx072, PMID: 28369176 PMC6433182

[30] Muldoon SF Soltesz I Cossart R. Spatially clustered neuronal assemblies comprise the microstructure of synchrony in chronically epileptic networks. Proc Natl Acad Sci U S A. (2013) 110:3567–72. doi: 10.1073/pnas.1216958110, PMID: 23401510 PMC3587208

[31] Ma HT Wu CH Wu JY. Initiation of spontaneous epileptiform events in the rat neocortex in vivo. J Neurophysiol. (2004) 91:934–45. doi: 10.1152/jn.00274.2003, PMID: 14534285 PMC2909741

[32] Ma HT Zhao MR Schwartz TH. Dynamic neurovascular coupling and uncoupling during ictal onset, propagation, and termination revealed by simultaneous in vivo optical imaging of neural activity and local blood volume. Cereb Cortex. (2013) 23:885–99. doi: 10.1093/cercor/bhs079, PMID: 22499798 PMC3593576

[33] Tsytsarev V Sopova JV Leonova EI Inyushin M Markina AA Chirinskaite AV. Neurophotonic methods in approach to in vivo animal epileptic models: advantages and limitations. Epilepsia. (2024) 65:600–14. doi: 10.1111/epi.17870, PMID: 38115808 PMC10948300

[34] Rodriguez EA Campbell RE Lin JY Lin MZ Miyawaki A Palmer AE. The growing and glowing toolbox of fluorescent and photoactive proteins. Trends Biochem Sci. (2017) 42:111–29. doi: 10.1016/j.tibs.2016.09.010, PMID: 27814948 PMC5272834

[35] Akerboom J Rivera JDV Guilbe MMR Malave ECA Hernandez HH Tian L. Crystal structures of the GCaMP calcium sensor reveal the mechanism of fluorescence signal change and aid rational design. J Biol Chem. (2009) 284:6455–64. doi: 10.1074/jbc.M807657200, PMID: 19098007 PMC2649101

[36] Nagai T Sawano A Park ES Miyawaki A. Circularly permuted green fluorescent proteins engineered to sense Ca2+. Proc Natl Acad Sci USA. (2001) 98:3197–202. doi: 10.1073/pnas.051636098, PMID: 11248055 PMC30630

[37] Dana H Sun Y Mohar B Hulse BK Kerlin AM Hasseman JP. High-performance calcium sensors for imaging activity in neuronal populations and microcompartments. Nat Methods. (2019) 16:649–57. doi: 10.1038/s41592-019-0435-6, PMID: 31209382

[38] Dana H Mohar B Sun Y Narayan S Gordus A Hasseman JP. Sensitive red protein calcium indicators for imaging neural activity. eLife. (2016) 5:24. doi: 10.7554/eLife.12727 PMC4846379 27011354

[39] Mohr MA Bushey D Aggarwal A Marvin JS Kim JJ Marquez EJ. jYCaMP: an optimized calcium indicator for two-photon imaging at fiber laser wavelengths. Nat Methods. (2020) 17:694–7. doi: 10.1038/s41592-020-0835-7, PMID: 32451475 PMC7335340

[40] Inoue M Takeuchi A Manita S Horigane S Sakamoto M Kawakami R. Rational engineering of XCaMPs, a multicolor GECI suite for in vivo imaging of complex brain circuit dynamics. Cell. (2019) 177:1346–1360.e24. doi: 10.1016/j.cell.2019.04.007, PMID: 31080068

[41] Qian Y Piatkevich KD Mc Larney B Abdelfattah AS Mehta S Murdock MH. A genetically encoded near-infrared fluorescent calcium ion indicator. Nat Methods. (2019) 16:171–4. doi: 10.1038/s41592-018-0294-6, PMID: 30664778 PMC6393164

[42] Sabatini BL Tian L. Imaging neurotransmitter and neuromodulator dynamics in vivo with genetically encoded indicators. Neuron. (2020) 108:17–32. doi: 10.1016/j.neuron.2020.09.036 33058762

[43] Berglund K Wen L Dunbar RL Feng GP Augustine GJ. Optogenetic visualization of presynaptic tonic inhibition of cerebellar parallel fibers. J Neurosci. (2016) 36:5709–23. doi: 10.1523/JNEUROSCI.4366-15.2016, PMID: 27225762 PMC4879193

[44] Sato SS Artoni P Landi S Cozzolino O Parra R Pracucci E. Simultaneous two-photon imaging of intracellular chloride concentration and pH in mouse pyramidal neurons in vivo. Proc Natl Acad Sci USA. (2017) 114:E8770–9. doi: 10.1073/pnas.1702861114 28973889 PMC5642681

[45] Bischof H Rehberg M Stryeck S Artinger K Eroglu E Waldeck-Weiermair M. Novel genetically encoded fluorescent probes enable real-time detection of potassium in vitro and in vivo. Nat Commun. (2017) 8:12. doi: 10.1038/s41467-017-01615-z 29127288 PMC5681659

[46] Shen Y Wu SY Rancic V Aggarwal A Qian Y Miyashita SI. Genetically encoded fluorescent indicators for imaging intracellular potassium ion concentration. Commun Biol. (2019) 2:10. doi: 10.1038/s42003-018-0269-2 30652129 PMC6331434

[47] Cabán CCT Yang MH Lai CX Yang LN Subach FV Smith BO. Tuning the sensitivity of genetically encoded fluorescent potassium indicators through structure-guided and genome mining strategies. ACS Sensors. (2022) 7:1336–46. doi: 10.1021/acssensors.1c02201 35427452 PMC9150168

[48] Rossano AJ Chouhan AK Macleod GT. Genetically encoded pH-indicators reveal activity-dependent cytosolic acidification of Drosophila motor nerve termini in vivo. J Physiol. (2013) 591:1691–706. doi: 10.1113/jphysiol.2012.248377, PMID: 23401611 PMC3624846

[49] Marvin JS Borghuis BG Tian L Cichon J Harnett MT Akerboom J. An optimized fluorescent probe for visualizing glutamate neurotransmission. Nat Methods. (2013) 10:162–70. doi: 10.1038/nmeth.2333, PMID: 23314171 PMC4469972

[50] Marvin JS Scholl B Wilson DE Podgorski K Kazemipour A Müller JA. Stability, affinity, and chromatic variants of the glutamate sensor iGluSnFR. Nat Methods. (2018) 15:936–9. doi: 10.1038/s41592-018-0171-3, PMID: 30377363 PMC6394230

[51] Marvin JS Shimoda Y Magloire V Leite M Kawashima T Jensen TP. A genetically encoded fluorescent sensor for in vivo imaging of GABA. Nat Methods. (2019) 16:763–70. doi: 10.1038/s41592-019-0471-2, PMID: 31308547

[52] Sun FM Zeng JZ Jing M Zhou JH Feng JS Owen SF. A genetically encoded fluorescent sensor enables rapid and specific detection of dopamine in flies, fish, and mice. Cell. (2018) 174:481–496.e19. doi: 10.1016/j.cell.2018.06.042, PMID: 30007419 PMC6092020

[53] Patriarchi T Cho JR Merten K Howe MW Marley A Xiong WH. Ultrafast neuronal imaging of dopamine dynamics with designed genetically encoded sensors. Science. (2018) 360:eaat4422. doi: 10.1126/science.aat4422 29853555 PMC6287765

[54] Deng F Wan JX Li GC Dong H Xia XJ Wang YP. Improved green and red GRAB sensors for monitoring spatiotemporal serotonin release in vivo. Nat Methods. (2024) 21:692–702. doi: 10.1038/s41592-024-02188-8, PMID: 38443508 PMC11377854

[55] Unger EK Keller JP Altermatt M Liang RQ Matsui A Dong CY. Directed evolution of a selective and sensitive serotonin sensor via machine learning. Cell. (2020) 183:1986–2002.e26. doi: 10.1016/j.cell.2020.11.040, PMID: 33333022 PMC8025677

[56] Dong A He KK Dudok B Farrell JS Guan WQ Liput DJ. A fluorescent sensor for spatiotemporally resolved imaging of endocannabinoid dynamics in vivo. Nat Biotechnol. (2022) 40:787–98. doi: 10.1038/s41587-021-01074-4, PMID: 34764491 PMC9091059

[57] Borden PM Zhang P Shivange AV Marvin JS Cichon J Dan C. A fast genetically encoded fluorescent sensor for faithful in vivo acetylcholine detection in mice, fish, worms and flies. bioRxiv. (2020) 2020.02.07.939504

[58] Lobas MA Tao R Nagai J Kronschläger MT Borden PM Marvin JS. A genetically encoded single-wavelength sensor for imaging cytosolic and cell surface ATP. Nat Commun. (2019) 10:13. doi: 10.1038/s41467-019-08441-5 30755613 PMC6372613

[59] Bando Y Sakamoto M Kim S Ayzenshtat I Yuste R. Comparative evaluation of genetically encoded voltage indicators. Cell Rep. (2019) 26:802–813.e4. doi: 10.1016/j.celrep.2018.12.088, PMID: 30650368 PMC7075032

[60] Liu ZH Lu XY Villette V Gou YY Colbert KL Lai SJ. Sustained deep-tissue voltage recording using a fast indicator evolved for two-photon microscopy. Cell. (2022) 185:3408–3425.e29. doi: 10.1016/j.cell.2022.07.013, PMID: 35985322 PMC9563101

[61] Gong YY Huang C Li JZ Grewe BF Zhang YP Eismann S. High-speed recording of neural spikes in awake mice and flies with a fluorescent voltage sensor. Science. (2015) 350:1361–6. doi: 10.1126/science.aab0810, PMID: 26586188 PMC4904846

[62] Adam Y Kim JJ Lou S Zhao YX Xie ME Brinks D. Voltage imaging and optogenetics reveal behaviour-dependent changes in hippocampal dynamics. Nature. (2019) 569:413–7. doi: 10.1038/s41586-019-1166-7, PMID: 31043747 PMC6613938

[63] Abdelfattah AS Kawashima T Singh A Novak O Liu H Shuai YC. Bright and photostable chemigenetic indicators for extended in vivo voltage imaging. Science. (2019) 365:699–704. doi: 10.1126/science.aav6416 31371562

[64] Benninger RKP Piston DW. Two-photon excitation microscopy for the study of living cells and tissues. Curr Protoc Cell Biol. (2013) Chapter 4:4.11.1–4.11.24. doi: 10.1002/0471143030.cb0411s59 PMC4004770 23728746

[65] Hillman EMC Voleti V Li WZ Yu H. Light-sheet microscopy in neuroscience In: Roska B Zoghbi HY, editors. Annual review of neuroscience, vol. 42. Palo Alto: Annual Reviews (2019). 295–313. 10.1146/annurev-neuro-070918-050357 PMC6800245 31283896

[66] Lawlor PN Goldberg EM. Dynamic fluorescence imaging in experimental epilepsy. Epilepsy Curr. (2022) 22:364–71. doi: 10.1177/15357597221113436, PMID: 36426179 PMC9661623

[67] Helmchen F Denk W. Deep tissue two-photon microscopy. Nat Methods. (2005) 2:932–40. doi: 10.1038/nmeth818 16299478

[68] Svoboda K Yasuda R. Principles of two-photon excitation microscopy and its applications to neuroscience. Neuron. (2006) 50:823–39. doi: 10.1016/j.neuron.2006.05.019, PMID: 16772166

[69] Ghosh KK Burns LD Cocker ED Nimmerjahn A Ziv Y El Gamal A. Miniaturized integration of a fluorescence microscope. Nat Methods. (2011) 8:871–8. doi: 10.1038/nmeth.1694, PMID: 21909102 PMC3810311

[70] Helmchen F Fee MS Tank DW Denk W. A miniature head-mounted two-photon microscope: high-resolution brain imaging in freely moving animals. Neuron. (2001) 31:903–12. doi: 10.1016/S0896-6273(01)00421-4, PMID: 11580892

[71] Zong WJ Wu RL Li ML Hu YH Li YJ Li JH. Fast high-resotution miniature two-photon microscopy for brain imaging in freely behaving mice. Nat Methods. (2017) 14:713–9. doi: 10.1038/nmeth.4305 28553965

[72] Muldoon SF Villette V Tressard T Malvache A Reichinnek S Bartolomei F. GABAergic inhibition shapes interictal dynamics in awake epileptic mice. Brain. (2015) 138:2875–90. doi: 10.1093/brain/awv227, PMID: 26280596

[73] de Curtis M Avanzini G. Interictal spikes in focal epileptogenesis. Prog Neurobiol. (2001) 63:541–67. doi: 10.1016/S0301-0082(00)00026-5 11164621

[74] Rossi LF Wykes RC Kullmann DM Carandini M. Focal cortical seizures start as standing waves and propagate respecting homotopic connectivity. Nat Commun. (2017) 8:1–11. doi: 10.1038/s41467-017-00159-6 28794407 PMC5550430

[75] Steinmetz NA Buetfering C Lecoq J Lee CR Peters AJ Jacobs EAK. Aberrant cortical activity in multiple GCaMP6-expressing transgenic mouse lines. eNeuro. (2017) 4:ENEURO.0207-17.2017. doi: 10.1523/ENEURO.0207-17.2017 PMC5604087 28932809

[76] Montgomery MK Kim SH Dovas A Zhao HZT Goldberg AR Xu WH. Glioma-induced alterations in neuronal activity and neurovascular coupling during disease progression. Cell Rep. (2020) 31:107500. doi: 10.1016/j.celrep.2020.03.064 32294436 PMC7443283

[77] Luo PJ Yang F Li J Niemeyer JE Zhan FR Estin J. Excitatory-inhibitory mismatch shapes node recruitment in an epileptic network. Epilepsia. (2023) 64:1939–50. doi: 10.1111/epi.17638, PMID: 37133275

[78] Cozzolino O Sicca F Paoli E Trovato F Santorelli FM Ratto GM. Evolution of epileptiform activity in zebrafish by statistical-based integration of electrophysiology and 2-photon ca<SUP>2+</SUP> imaging. Cells. (2020) 9:19. doi: 10.3390/cells9030769 PMC7140665 32245158

[79] Liu J Salvati KA Baraban SC. In vivo calcium imaging reveals disordered interictal network dynamics in epileptic stxbp1b zebrafish. iScience. (2021) 24:102558. doi: 10.1016/j.isci.2021.102558 34142057 PMC8184515

[80] Niemeyer JE Gadamsetty P Chun C Sylvester S Lucas JP Ma HC. Seizures initiate in zones of relative hyperexcitation in a zebrafish epilepsy model. Brain. (2022) 145:2347–60. doi: 10.1093/brain/awac073, PMID: 35196385 PMC9612797

[81] Liu J Baraban SC. Network properties revealed during multi-scale calcium imaging of seizure activity in zebrafish. eNeuro. (2019) 6:ENEURO.0041-19.2019. doi: 10.1523/ENEURO.0041-19.2019 PMC6424556 30895220

[82] Masala N Pofahl M Haubrich AN Islam KUS Nikbakht N Pasdarnavab M. Targeting aberrant dendritic integration to treat cognitive comorbidities of epilepsy. Brain. (2023) 146:2399–417. doi: 10.1093/brain/awac455, PMID: 36448426 PMC10232249

[83] Sparks FT Liao Z Li W Grosmark A Soltesz I Losonczy A. Hippocampal adult-born granule cells drive network activity in a mouse model of chronic temporal lobe epilepsy. Nat Commun. (2020) 11:13. doi: 10.1038/s41467-020-19969-2 33262339 PMC7708476

[84] Hadjiabadi D Lovett-Barron M Raikov IG Sparks FT Liao ZR Baraban SC. Maximally selective single-cell target for circuit control in epilepsy models. Neuron. (2021) 109:2556–2572.e6. doi: 10.1016/j.neuron.2021.06.007, PMID: 34197732 PMC8448204

[85] Jamiolkowski RM Nguyen QA Farrell JS McGinn RJ Hartmann DA Nirschl JJ. The fasciola cinereum of the hippocampal tail as an interventional target in epilepsy. Nat Med. (2024) 30:1292–9. doi: 10.1038/s41591-024-02924-9 38632391 PMC11108783

[86] Heuser K Nome CG Pettersen KH Åbjorsbråten KS Jensen V Tang WN. Ca2+ signals in astrocytes facilitate spread of epileptiform activity. Cereb Cortex. (2018) 28:4036–48. doi: 10.1093/cercor/bhy196, PMID: 30169757 PMC6188565

[87] Petrucco L Pracucci E Brondi M Ratto GM Landi S. Epileptiform activity in the mouse visual cortex interferes with cortical processing in connected areas. Sci Rep. (2017) 7:40054. doi: 10.1038/srep40054, PMID: 28071688 PMC5223162

[88] Li M Eltabbal M Tran HD Kuhn B. Scn2a insufficiency alters spontaneous neuronal Ca2+ activity in somatosensory cortex wakefulness. iScience. (2023) 26:108138. doi: 10.1016/j.isci.2023.108138 37876801 PMC10590963

[89] Aeed F Shnitzer T Talmon R Schiller Y. Layer- and cell-specific recruitment dynamics during epileptic seizures in vivo. Ann Neurol. (2020) 87:97–115. doi: 10.1002/ana.25628, PMID: 31657482

[90] Bando Y Wenzel M Yuste R. Simultaneous two-photon imaging of action potentials and subthreshold inputs in vivo. Nat Commun. (2021) 12:12. doi: 10.1038/s41467-021-27444-9 34893595 PMC8664861

[91] Shimoda Y Leite M Graham RT Marvin JS Hasseman J Kolb I. Extracellular glutamate and GABA transients at the transition from interictal spiking to seizures. Brain. (2024) 147:1011–24. doi: 10.1093/brain/awad336, PMID: 37787057 PMC10907087

[92] Magloire V Mercier MS Kullmann DM Pavlov I. GABAergic interneurons in seizures: investigating causality with Optogenetics. Neuroscientist. (2019) 25:344–58. doi: 10.1177/1073858418805002 30317911 PMC6745605

[93] Li J Yang F Zhan FR Estin J Iyer A Zhao MR. Mesoscopic mapping of hemodynamic responses and neuronal activity during pharmacologically induced interictal spikes in awake and anesthetized mice. J Cereb Blood Flow Metab. (2024) 44:911–24. doi: 10.1177/0271678X241226742 38230631 PMC11318398

[94] Shuman T Aharoni D Cai DJ Lee CR Chavlis S Page-Harley L. Breakdown of spatial coding and interneuron synchronization in epileptic mice. Nat Neurosci. (2020) 23:229–38. doi: 10.1038/s41593-019-0559-0, PMID: 31907437 PMC7259114

[95] Wenzel M Hamm JP Peterka DS Yuste R. Acute focal seizures start as local synchronizations of neuronal ensembles. J Neurosci. (2019) 39:8562–75. doi: 10.1523/JNEUROSCI.3176-18.2019 31427393 PMC6807279

[96] Brenet A Hassan-Abdi R Somkhit J Yanicostas C Soussi-Yanicostas N. Defective excitatory/inhibitory synaptic balance and increased neuron apoptosis in a zebrafish model of Dravet syndrome. Cells. (2019) 8:13. doi: 10.3390/cells8101199 PMC6829503 31590334

[97] Liao MJ Kundap U Rosch RE Burrows DRW Meyer MP Bencheikh BOA. Targeted knockout of GABA-A receptor gamma 2 subunit provokes transient light-induced reflex seizures in zebrafish larvae. Dis Model Mech. (2019) 12:11. doi: 10.1242/dmm.040782 PMC6899022 31582559

[98] Verdugo CD Myren-Svelstad S Aydin E Van Hoeymissen E Deneubourg C Vanderhaeghe S. Glia-neuron interactions underlie state transitions to generalized seizures. Nat Commun. (2019) 10:13. doi: 10.1038/s41467-019-11739-z 31444362 PMC6707163

[99] Turrini L Sorelli M de Vito G Credi C Tiso N Vanzi F. Multimodal characterization of seizures in zebrafish larvae. Biomedicines. (2022) 10:16. doi: 10.3390/biomedicines10050951 PMC9139036 35625689

[100] Hotz AL Jamali A Rieser NN Niklaus S Aydin E Myren-Svelstad S. Loss of glutamate transporter eaat2a leads to aberrant neuronal excitability, recurrent epileptic seizures, and basal hypoactivity. Glia. (2022) 70:196–214. doi: 10.1002/glia.24106, PMID: 34716961 PMC9297858

[101] Myren-Svelstad S Jamali A Ophus SS D'Gama PP Ostenrath AM Mutlu AK. Elevated photic response is followed by a rapid decay and depressed state in ictogenic networks. Epilepsia. (2022) 63:2543–60. doi: 10.1111/epi.17380, PMID: 36222083 PMC9804334

[102] Tran CH Vaiana M Nakuci J Somarowthu A Goff KM Goldstein N. Interneuron desynchronization precedes seizures in a mouse model of Dravet syndrome. J Neurosci. (2020) 40:2764–75. doi: 10.1523/JNEUROSCI.2370-19.2020, PMID: 32102923 PMC7096149

[103] Lim HK You N Bae S Kang BM Shon YM Kim SG. Differential contribution of excitatory and inhibitory neurons in shaping neurovascular coupling in different epileptic neural states. J Cereb Blood Flow Metab. (2021) 41:1145–61. doi: 10.1177/0271678X20934071, PMID: 32669018 PMC8054729

[104] Rosch RE Hunter PR Baldeweg T Friston KJ Meyer MP. Calcium imaging and dynamic causal modelling reveal brain-wide changes in effective connectivity and synaptic dynamics during epileptic seizures. PLoS Comput Biol. (2018) 14:23. doi: 10.1371/journal.pcbi.1006375 PMC6124808 30138336

[105] Burrows DRW Diana G Pimpel B Moeller F Richardson MP Bassett DS. Microscale neuronal activity collectively drives chaotic and inflexible dynamics at the macroscale in seizures. J Neurosci. (2023) 43:3259–83. doi: 10.1523/JNEUROSCI.0171-22.2023, PMID: 37019622 PMC7614507

[106] Zhang J Liu XJ Xu WJ Luo WH Li M Chu FB. Stretchable transparent electrode arrays for simultaneous electrical and optical interrogation of neural circuits in vivo. Nano Lett. (2018) 18:2903–11. doi: 10.1021/acs.nanolett.8b00087, PMID: 29608857

[107] Driscoll N Rosch RE Murphy BB Ashourvan A Vishnubhotla R Dickens OO. Multimodal in vivo recording using transparent graphene microelectrodes illuminates spatiotemporal seizure dynamics at the microscale. Commun Biol. (2021) 4:14. doi: 10.1038/s42003-021-01670-9 33514839 PMC7846732

[108] Mulcahey PJ Chen YZ Driscoll N Murphy BB Dickens OO Johnson ATC. Multimodal, multiscale insights into hippocampal seizures enabled by transparent, graphene-based microelectrode arrays. eNeuro. (2022) 9:ENEURO.0386-21.2022. doi: 10.1523/ENEURO.0386-21.2022 PMC9087744 35470227

[109] Zhang ZR Jiang SH Shi KB Li Y Jin WN Liu Q. Visualizing seizure propagation in freely-moving mice via miniature two-photon microscopy. Neurosci Bull. (2022) 38:1593–7. doi: 10.1007/s12264-022-00947-1, PMID: 36161581 PMC9723026

[110] Wong JC Grieco SF Dutt K Chen LJ Thelin JT Inglis GAS. Autistic-like behavior, spontaneous seizures, and increased neuronal excitability in a <i>Scn8a</i> mouse model. Neuropsychopharmacology. (2021) 46:2011–20. doi: 10.1038/s41386-021-00985-9, PMID: 33658654 PMC8429750

[111] Stern MA Cole ER Gross RE Berglund K. Seizure event detection using intravital two-photon calcium imaging data. Neurophotonics. (2024) 11:024202. doi: 10.1117/1.NPh.11.2.024202, PMID: 38274784 PMC10809036

[112] Jayant K Wenzel M Bando Y Hamm JP Mandriota N Rabinowitz JH. Flexible Nanopipettes for minimally invasive intracellular electrophysiology in vivo. Cell Rep. (2019) 26:266–278.e5. doi: 10.1016/j.celrep.2018.12.019, PMID: 30605681 PMC7263204

[113] Wenzel M Hamm JP Peterka DS Yuste R. Reliable and elastic propagation of cortical seizures in vivo. Cell Rep. (2017) 19:2681–93. doi: 10.1016/j.celrep.2017.05.090, PMID: 28658617 PMC5551439

[114] ShahPTValianteTAPackerAM. Highly local activation of inhibition at the seizure wavefront in vivo. Cell Rep. (2024) 43:114189. doi: 10.1016/j.celrep.2024.114189, PMID: 38703365

[115] Somarowthu A Goff KM Goldberg EM. Two-photon calcium imaging of seizures in awake, head-fixed mice. Cell Calcium. (2021) 96:102380. doi: 10.1016/j.ceca.2021.102380 33676317 PMC8187286

[116] Yang F Li J Song Y Zhao MR Niemeyer JE Luo PJ. Mesoscopic mapping of ictal neurovascular coupling in awake behaving mice using optical spectroscopy and genetically encoded calcium indicators. Front Neurosci. (2021) 15:704834. doi: 10.3389/fnins.2021.704834 34366781 PMC8343016

[117] Hatcher A Yu K Meyer J Aiba I Deneen B Noebels JL. Pathogenesis of peritumoral hyperexcitability in an immunocompetent CRISPR-based glioblastoma model. J Clin Invest. (2020) 130:2286–300. doi: 10.1172/JCI133316, PMID: 32250339 PMC7190940

[118] de Vito G Turrini L Müllenbroich C Ricci P Sancataldo G Mazzamuto G. Fast whole-brain imaging of seizures in zebrafish larvae by two-photon light-sheet microscopy. Biomed Opt Express. (2022) 13:1516–36. doi: 10.1364/BOE.434146, PMID: 35414999 PMC8973167

[119] Özsoy Ç Hotz AL Rieser NN Chen ZY Deán-Ben XL Neuhauss SCF. Volumetric optoacoustic neurobehavioral tracking of epileptic seizures in freely-swimming zebrafish larvae. Front Mol Neurosci. (2022) 15:9. doi: 10.3389/fnmol.2022.1004518 PMC9514119 36176960

[120] Turrini L Fornetto C Marchetto G Müllenbroich MC Tiso N Vettori A. Optical mapping of neuronal activity during seizures in zebrafish. Sci Rep. (2017) 7:12. doi: 10.1038/s41598-017-03087-z 28596596 PMC5465210

[121] Winter MJ Windell D Metz J Matthews P Pinion J Brown JT. 4-dimensional functional profiling in the convulsant-treated larval zebrafish brain. Sci Rep. (2017) 7:16. doi: 10.1038/s41598-017-06646-6 28747660 PMC5529444

[122] Meyer J Maheshwari A Noebels J Smirnakis S. Asynchronous suppression of visual cortex during absence seizures in stargazer mice. Nat Commun. (2018) 9:9. doi: 10.1038/s41467-018-04349-8 29769525 PMC5955878

[123] Berdyyeva TK Frady EP Nassi JJ Aluisio L Cherkas Y Otte S. Direct imaging of hippocampal epileptiform calcium motifs following Kainic acid Administration in Freely Behaving Mice. Front Neurosci. (2016) 10:53. doi: 10.3389/fnins.2016.00053 26973444 PMC4770289

[124] Farrell JS Colangeli R Dudok B Wolff MD Nguyen SL Jackson J. In vivo assessment of mechanisms underlying the neurovascular basis of postictal amnesia. Sci Rep. (2020) 10:13. doi: 10.1038/s41598-020-71935-6 32929133 PMC7490395

[125] Lau LA Zhao Z Gomperts SN Staley KJ Lillis KP. Cellular resolution contributions to ictal population signals. Epilepsia. (2024) 65:2165–78. doi: 10.1111/epi.17983 38752861 PMC11251866

[126] Farrell JS Colangeli R Dong A George AG Addo-Osafo K Kingsley PJ. In vivo endocannabinoid dynamics at the timescale of physiological and pathological neural activity. Neuron. (2021) 109:2398–2403.e4. doi: 10.1016/j.neuron.2021.05.026, PMID: 34352214 PMC8351909

[127] Nguyen QA Klein PM Xie C Benthall KN Iafrati J Homidan J. Acetylcholine receptor based chemogenetics engineered for neuronal inhibition and seizure control assessed in mice. Nat Commun. (2024) 15:13. doi: 10.1038/s41467-024-44853-8 38238329 PMC10796428

[128] Enger R Tang WN Vindedal GF Jensen V Helm PJ Sprengel R. Dynamics of ionic shifts in cortical spreading depression. Cereb Cortex. (2015) 25:4469–76. doi: 10.1093/cercor/bhv054, PMID: 25840424 PMC4816793

[129] Tamim I Chung DY de Morais AL Loonen ICM Qin T Misra A. Spreading depression as an innate antiseizure mechanism. Nat Commun. (2021) 12:1–15. doi: 10.1038/s41467-021-22464-x 33850125 PMC8044138

[130] Dreier JP Major S Pannek HW Woitzik J Scheel M Wiesenthal D. Spreading convulsions, spreading depolarization and epileptogenesis in human cerebral cortex. Brain. (2012) 135:259–75. doi: 10.1093/brain/awr303, PMID: 22120143 PMC3267981

[131] Tran CH George AG Teskey GC Gordon GR. Seizures elevate gliovascular unit Ca2+ and cause sustained vasoconstriction. JCI Insight. (2020) 5:12. doi: 10.1172/jci.insight.136469 PMC7566700 33004688

[132] Schevon CA Goodman RR McKhann G Emerson RG. Propagation of epileptiform activity on a submillimeter scale. J Clin Neurophysiol. (2010) 27:406–11. doi: 10.1097/WNP.0b013e3181fdf8a1, PMID: 21076338 PMC3039548

[133] Stead M Bower M Brinkmann BH Lee K Marsh WR Meyer FB. Microseizures and the spatiotemporal scales of human partial epilepsy. Brain. (2010) 133:2789–97. doi: 10.1093/brain/awq190, PMID: 20685804 PMC2929333

[134] Prince DA Wilder BJ. Control mechanisms in cortical epileptogenic foci - "surround" inhibition. Arch Neurol. (1967) 16:194–202. doi: 10.1001/archneur.1967.00470200082007, PMID: 6018049

[135] Khan L van Lanen R Hoogland G Schijns O Rijkers K Kapsokalyvas D. Two-photon imaging to unravel the Pathomechanisms associated with epileptic seizures: a review. Appl Sci. (2021) 11:24. doi: 10.3390/app11052404

[136] Lieb A Qiu YC Dixon CL Heller JP Walker MC Schorge S. Biochemical autoregulatory gene therapy for focal epilepsy. Nat Med. (2018) 24:1324–9. doi: 10.1038/s41591-018-0103-x, PMID: 29988123 PMC6152911

[137] Kim TH Zhang YP Lecoq J Jung JC Li J Zeng HK. Long-term optical access to an estimated one million neurons in the live mouse cortex. Cell Rep. (2016) 17:3385–94. doi: 10.1016/j.celrep.2016.12.004, PMID: 28009304 PMC5459490

[138] Ichimura T Kakizuka T Horikawa K Seiriki K Kasai A Hashimoto H. Exploring rare cellular activity in more than one million cells by a transscale scope. Sci Rep. (2021) 11:16. doi: 10.1038/s41598-021-95930-7 34400683 PMC8368064

[139] Horton NG Wang K Kobat D Clark CG Wise FW Schaffer CB. In vivo three-photon microscopy of subcortical structures within an intact mouse brain. Nat Photonics. (2013) 7:205–9. doi: 10.1038/nphoton.2012.336, PMID: 24353743 PMC3864872

[140] Zong WJ Obenhaus HA Skytoen ER Eneqvist H de Jong NL Vale R. Large-scale two-photon calcium imaging in freely moving mice. Cell. (2022) 185:1240–1256.e30. doi: 10.1016/j.cell.2022.02.017, PMID: 35305313 PMC8970296

